# Short- and Long-Term Nutritional Status in Children and Adolescents with Celiac Disease Following a Gluten-Free Diet: A Systematic Review

**DOI:** 10.3390/nu17030487

**Published:** 2025-01-29

**Authors:** Maria Papoutsaki, Christina N. Katsagoni, Alexandra Papadopoulou

**Affiliations:** 1 Department of Clinical Nutrition, Agia Sofia Children’s Hospital, 11527 Athens, Greece; marypap99@gmail.com (M.P.); christina.katsagoni@gmail.com (C.N.K.); 2 Department of Nutrition & Dietetics, School of Health Sciences and Education, Harokopio University, 11527 Athens, Greece; 3 Division of Gastroenterology and Hepatology, First Department of Pediatrics, University of Athens, 11527 Athens, Greece

**Keywords:** celiac disease, gluten-free diet, nutritional deficiencies, pediatric

## Abstract

Background/Objectives: Numerous studies have highlighted the nutritional imbalances that are commonly observed in children and adolescents diagnosed with celiac disease (CD) who follow a gluten-free diet (GFD). However, the development and timeline of these nutritional deficiencies remain unclear. The aim of the present study is to investigate the short-term (≥6 months to <12 months) and long-term (≥12 months) association between adherence to a GFD and nutrient intake as well as micronutrient blood status in children and adolescents aged from 0 to 18 years with CD. Methods: A systematic review was conducted in PubMed and Scopus for observational studies published up to June 2024. Results: A total of 15 studies (case–control, cross-sectional, and prospective studies) with 2004 children and adolescents were included. Their quality was assessed using the ROBINS-E tool. Despite the lack of high-quality data and the heterogeneity of the methods used in the included studies, the results of the cross-sectional/case–control studies show that, in the short term, children and adolescents with CD consumed excessive amounts of protein and carbohydrates compared to controls. After long-term adherence to a GFD, significant changes in the diets of children and adolescents with CD persisted. Fat intake was higher, while protein intake remained excessive compared to controls. Based on prospective studies, vitamin C and iodine intake improved both in the short and long term after adherence to a GFD. However, most other nutrients either remain inadequate or continue to decline, indicating that it is difficult to meet nutrient requirements despite dietary adjustments. Conclusions: Gaps in adherence to dietary recommendations appear to be widespread in children and adolescents with CD, emphasizing the need for improved diet quality and regular monitoring.

## 1. Introduction

Celiac disease (CD) is a chronic multi-organ autoimmune disorder that primarily affects the small intestine in genetically predisposed individuals, including both children and adults [1]. It is marked by a range of gluten-dependent symptoms, the presence of specific antibodies, HLA-DQ2 or HLA-DQ8 genetic markers, and intestinal damage. The antibodies specific to CD include autoantibodies against tissue transglutaminase (TG2), such as endomysial antibodies (EMA), and antibodies against deamidated gliadin peptides (DGP) [2]. The overall global prevalence of CD is 1.4% based on positive blood tests and 0.7% based on biopsy results, with the disease being approximately twice as common in children as in adults [3]. Children with Type 1 diabetes [4], autoimmune diseases [5], and conditions like trisomy 21 [6], Turner syndrome, Williams syndrome [3], selective IgA deficiency, lupus, and juvenile chronic arthritis disease [7] are at higher risk for CD [1,8].

Up until today, the only treatment for CD is the strict adherence to a gluten-free diet (GFD) [9,10], which requires the complete elimination of wheat [11], rye, and barley [12], while elimination of oats is not so straightforward [13]. Several nutritional deficiencies and imbalances have been pointed out in the literature in children and adolescents with CD, which may be directly related to the disease itself (i.e., time before diagnosis) and/or being a consequence of a GFD, the location and extent of small bowel damage, or the severity of malabsorption [14,15,16]. However, these imbalances can also occur in patients who have achieved histologically confirmed remission, which means that they cannot be attributed to the continued consumption of gluten or the resulting malabsorption [17,18,19]. Other factors, such as the quality of commercially available gluten-free products, patients’ dietary habits, and changes in their daily food intake, are considered to influence the nutritional adequacy of patients’ diets [17].

Indeed, compared to gluten-containing diets, GFD is characterized by the low intake of cereals, fruits, and vegetables and the excessive consumption of meat and gluten-free products [20]. Moreover, three recent systematic reviews [17,18,19] have shown that children, regardless of whether they are on a GFD or not, tend to consume excessive amounts of fat and inadequate levels of fiber, iron vitamin D, calcium, folate, magnesium, zinc, and selenium, with those following a GFD being at a greater risk. Additionally, it has been found that the majority of the patients (over 80%) consume gluten-free products two to three times a day [18,21,22]. Gluten-free products are of relatively low quality as they are deficient in protein, fiber, and essential micronutrients, which raises concerns about their suitability as part of a balanced GFD [23,24,25]. At the same time, the elevated caloric, sugar, and fat content of the highly processed gluten-free products [21], especially gluten-free snacks [26], together with the enhanced intestinal absorption resulting from mucosal healing in the setting of a GFD, may contribute to excessive calorie intake [18,27], underscoring the need for careful nutritional management to prevent growth and weight issues in children with CD.

As far as the effects on the child’s physical development are concerned, it is assumed that overweight or obesity is more common than previously thought, which calls into question the long-held view of malnutrition in this group [18]. Indeed, a meta-analysis by Diamanti et al. [28] pointed out that overweight is identified in 8.8%–20.8% of children with CD at diagnosis, while obesity affects 0%–6% of patients. These rates increased from 9.4% to 21% for overweight and from 0% to 8.8% for obesity among patients with CD following a GFD. In addition to the previous results, Giuseppe et al. [29] showed that the prevalence of overweight or obesity in school-age children (6–17 years) with CD ranged between 3.5% and 20%. However, no significant associations were reported in the meta-analysis by Barone et al. [30], since the results showed that GFD did not increase the risk of overweight/obesity in children, suggesting that more long-term studies should be conducted in this population.

Therefore, although systematic reviews have highlighted the nutritional deficits and imbalances associated with a GFD, there is an obvious need for a comprehensive analysis of how these nutritional deficits evolve over time and which specific deficits may become more or less pronounced with prolonged adherence to a GFD. Particular attention should be given to weight changes, as unbalanced calorie intake and nutrient absorption may not only lead to undernutrition, but also contribute to the risk of overweight and obesity. Thus, the primary aim of the present systematic review was to investigate both short-term (from ≥6 months to <12 months) and long-term (≥12 months) nutrient intake (i.e., macro- and micronutrient intake) and micronutrient blood status amongst children and adolescents aged from 0 to 18 years with CD after following a GFD. Secondary objectives included a wide range of health outcomes in children and adolescents with CD, both short and long term, including anthropometric measurements, body composition assessments, and dietary habits.

## 2. Materials and Methods

### 2.1. Data Sources

A comprehensive literature search was conducted up to 30 June 2024, utilizing specific keywords in the databases of the US National Library of Medicine (PubMed.gov, accessed on 30 June 2024) and Scopus (www.scopus.com, accessed on 30 June 2024). Two independent researchers (M.P. and C.N.K.) meticulously reviewed and identified all pertinent publications. The Medical Subject Heading (MeSH) keywords used were as follows: celiac disease, gluten, gluten-free diet, GFD, anthropometry, weight, height, BMI, nutrient deficiency, vitamin and/or mineral deficiency, macronutrient, micronutrient, nutritional status, diet quality, dietary therapy, children, adolescents, pediatric, kids, child, youth, short-term, long-term, 6 months, and 12 months, as well as combinations of the above. The reference list of the retrieved articles and reviews were examined to identify additional relevant studies. Studies were evaluated using a hierarchical approach, based on the title, the abstract, and the full text of each study. For the purposes of this review, all relevant studies published from 1998 onward were taken into account. The present study follows the Preferred Reporting Items for Systematic Review and Meta-Analyses (PRISMA) criteria [31].

This systematic review was also carried out following a protocol, available on the Web at http://www.crd.york.ac.uk/PROSPERO/, the registration number of which is CRD42024619926 (registered on 10 December 2024).

### 2.2. Inclusion and Exclusion Criteria

Eligible studies were observational studies (i.e., case–control, cross-sectional, and prospective cohort studies) involving children and adolescents aged from 0 to 18 years diagnosed with CD who had either been following a GFD for a minimum of 6 months or up to or over 12 months post-diagnosis. This systematic review focused exclusively on observational studies of patients in whom the diagnosis of CD was confirmed using international criteria. Studies investigating the intake of energy, macronutrients (protein, carbohydrate, fat, and fiber), and micronutrients (fat- and water-soluble vitamins, and essential and trace elements) were included. The search was limited to English language studies that were available in full text.

The main exclusion criteria were studies with adult or mixed populations in which data for children and adolescents were not reported separately, studies without a clear time frame for adherence to the GFD, and studies in which the GFD was not consistently followed by participants. Animal studies, systematic reviews, and randomized controlled trials were also excluded from the present study.

### 2.3. Data Extraction

The review process for all relevant studies was conducted using the Rayyan web tool. After duplicate removal, the studies underwent a two-stage screening process to assess eligibility. First, researchers (M.P. and C.N.K.) independently conducted a title and abstract screening. Articles excluded at this stage were based on their titles and abstracts. The remaining studies were then subjected to a full-text review by both researchers, applying the eligibility criteria to finalize the selection of studies. Any disagreements were resolved by the third author (A.P.). For all studies, we extracted information on authors, journal and year of publication, methods (study design), inclusion and exclusion criteria, study sample, patient population characteristics (i.e., number, age, and sex), CD diagnosis, adherence to a GFD, duration of the GFD, outcomes measured, tools used to measure the outcomes, and the study results.

### 2.4. Outcome Measures

The primary outcomes of the included studies were energy, macro- and micronutrient intake, and blood micronutrient levels in children and adolescents aged from 0 to 18 years with CD on a GFD, both with short-term (≥6 months to <12 months) and long-term (≥12 months) adherence.

Secondary outcomes were anthropometric measurements (i.e., weight, body weight z-score, height, height z-score, body mass index (BMI), BMI z-score, BMI for age, height for age, body fat, fat mass, and muscle mass), bone mineral density (BMD), and dietary habits (i.e., food groups, gluten-free products).

### 2.5. Risk of Bias

The quality assessment of epidemiological studies was conducted with the Risk Of Bias In Non-randomized Studies-of Exposures (ROBINS-E) tool (https://www.riskofbias.info/welcome/robins-e-tool) [32]. The ROBINS-E tool comprises the following domains: (1) bias due to confounding, (2) bias arising from measurement of the exposure, (3) bias in selection of participants into the study (or into the analysis), (4) bias due to post-exposure interventions, (5) bias due to missing data, (6) bias arising from measurement of the outcome, and (7) bias in selection of the reported result. Each domain and the overall study are assessed as “low risk”, “some concerns”, “high risk”, or “very high risk”. The overall judgment of the quality of non-randomized clinical trials was based on the worst level of bias that each study received for a particular domain.

## 3. Results

### 3.1. Study Selection and Quality Assessment

**Figure 1:**
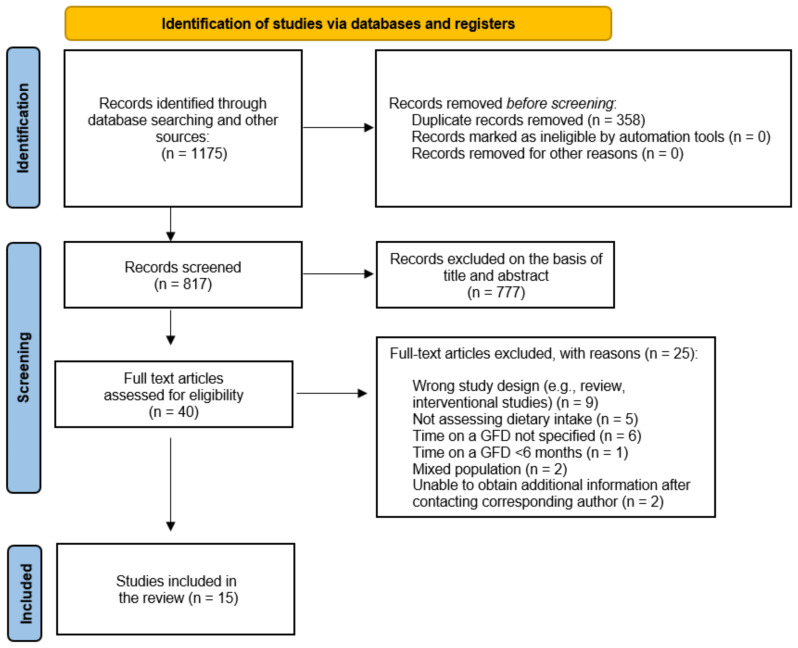
Flowchart of the systematic review based on PRISMA guidelines.

The initial search yielded 1,175 results in the database. Of these, 358 duplicate entries were eliminated, leaving 817 unique studies for screening. A total of 40 full-text studies were assessed in detail for their suitability. Of these, 15 studies were included in the assessment after meeting all inclusion and exclusion criteria. The flowchart is shown in Figure 1.

Findings with regard to the quality of the eligible studies are shown in Table 1. Based on ROBINS-E tool, 2/15 showed “low” risk of bias, 3/15 showed “some concerns” risk of bias, 3/15 showed “high” risk of bias, and 7/15 showed “very high” risk of bias. Most of the studies had a “very high” risk of bias, mainly since data on exposures derived from self-reported questionnaires or food diaries, often completed by children and their parents. Also, some studies did not screen adherence to GFD using serological markers.

### 3.2. Study Characteristics

Of the fifteen included studies, five were conducted in Italy [35,36,37,40,46], three in Spain [22,39,42], three in Poland [38,43,45], one in Turkey [34], one in Croatia [44], one in Canada [41], and one in Saudi Arabia [33]. The sample size of the included studies varied from 18 [46] to 590 [44] patients, with the overall analysis comprising 2004 participants (1053 children and adolescents with CD and 951 healthy participants). Amongst the 15 studies reviewed, conducted from 2003 [43] to 2022 [33,44,45], more than half (9 out of 15, or 60%) were published within the last five years, highlighting that the analysis incorporates a substantial portion of the most recent research in this field. All but two studies [22,33] recruited children and adolescents from clinics and hospitals. One study [33] utilized a database from the General Nutrition Department at the Saudi Ministry of Health, including participants enrolled in a GFD program, while another [22] recruited patients through the Celiac and Gluten Sensitive Association in Madrid, Spain [22], whereas healthy controls (HCs) in the same study were recruited from the general population [22].

Of the total, 9/15 [33,34,35,38,40,42,43,44,46] studies used the European Society for Paediatric Gastroenterology, Hepatology, and Nutrition guidelines (ESPGHAN) for celiac disease diagnosis [33,34,35,38,40,42,43,44,46]. Specifically, two studies [43,46] used the criteria published in 1990, three studies [38,40,42] confirmed CD using the criteria from 2012, and one study [44] followed the 2020 guidelines. Additionally, two research groups, Balamtekin et al. [34] and Ferrara et al. [35], reported that the diagnosis was made based on the ESPGHAN criteria, but the publication date was not specified. The research teams of Forchielli et al. [36,37] and Mager et al. [41] applied Marsh (1992) criteria. Finally, four studies [22,33,39,45] did not provide information regarding the diagnostic criteria for CD.

Only 9/15 studies assessed adherence to the GFD, while the methods and combinations used to assess adherence to the GFD varied from study to study. The majority of studies used serological markers, including immunoglobulin A anti-tissue transglutaminase antibodies (IgA-tTG) [22,36,37,41,46], anti-transglutaminase deamidated gliadin peptide (DGP) antibodies [36,37], antigliadin [46], and antiendomysium (EMA) [34,46] levels. Balamtekin et al. [34] used EMA IgA antibodies together with dietary history for the assessment of GFD adherence, while Rujner et al. [43] included mucosal biopsy evaluation as well. One study [40] assessed adherence to the GFD via serological markers, but did not specify which markers were used. In a study by Szaflarska-Popławska et al. [45] adherence was confirmed through evidence of clinical remission and exclusion of gluten transgressions based on detailed dietary interview by an experienced gastroenterologist. Notably, six studies did not assess adherence to a GFD [33,35,38,39,42,44].

In most studies included in the present systematic review, the FFQ was the primary assessment tool of energy, macronutrient, and micronutrient intake [22,35,36,37,39,42,43,46]. Among these, three studies incorporated three 24 h recalls alongside the FFQ [22,39,42], two supplemented the FFQ with a single 24 h recall [24,26], while one combined FFQ with food diaries [27]. Alternative tools for dietary assessment included food diaries [38,40], 3-day food records [37,39,40], and three 24 h recalls, covering two weekdays and one weekend day [42].

### 3.3. Associations of GFD with Energy and Macronutrient Intake

Methodological discrepancies were noted both across and within the short-term and long-term dietary patterns categories, particularly in terms of the comparison group. In total, 2/15 studies [33,36] studies evaluated energy and macronutrient intake in children with CD, comparing their dietary patterns with established dietary guidelines, 1/15 studies [39] combined established dietary guidelines with a simulated gluten-containing diet, and 4/15 studies [34,35,42,43] compared the dietary intake of children and adolescents with CD to that of HCs. In addition, in 5/15 studies [22,40,44,45,46] the values were compared to those in participants without CD and the national dietary reference intake (DRI) standards. Finally, 3/15 studies [37,38,41] conducted a comprehensive analysis of how nutritional deficiencies evolve from time of diagnosis to short- and long-term adherence to a GFD.

The main characteristics of the studies and a summary of the main findings regarding energy and macronutrient intake are shown in Table 2.

Short-term associations

As detailed in Table 2, only three studies [33,42,46] (i.e., one cross-sectional and two cross-sectional/case–control studies) evaluated the nutrient intake of children and adolescents with CD following a short-term GFD, showing differences in energy and macronutrient intake, which depend on the comparison group and the reference dietary patterns.

Allowaymi et al. [33] evaluated the nutrient intake of 66 Saudi children with CD following the Ministry of Health’s GFD program for over 6 months. The mean values of children’s nutrient intake were compared with the DRI and yielded contradictory results: While energy intake was significantly lower than the DRI, carbohydrate and protein intake was significantly higher, whereas no differences were found in fat intake after 6 months on a GFD. In addition, fiber intake was lower than the DRI. However, Nestares et al. [42] did not report any significant difference for total energy and macronutrient intake between 68 children with CD following a GFD for more than 6 months compared to HCs.

A study by Zuccotti et al. [46] included 18 children with CD on a short-term GFD to 18 non-celiac controls. The study examined energy consumption and macronutrient distribution in children with CD and HCs, and also compared the results to the Italian recommended daily intake (LARN). In contrast to the study by Allowaymi et al. [33], researchers showed that children with CD had a significantly higher median daily energy intake compared to the HCs. The LARN recommendation of 12% protein energy intake was exceeded by both groups of children with CD or without CD. Children with CD consumed more protein overall than HCs, even though the percentage of calories from protein did not differ significantly across groups. In addition, median carbohydrate intake was also significantly higher in patients with CD and, unlike controls, it was aligned with LARN recommendations. Energy derived from fat was significantly lower in children with CD, although fat intake in both groups exceeded LARN recommendations. Finally, there were no significant differences in the intake of fiber, saturated (SFA), monounsaturated (MUFA), or polyunsaturated fats (PUFA) between the two groups.

Long-term associations

Although numerous studies have investigated the effects of long-term adherence to a GFD on nutrient intake, their results have been contradictory.

Specifically, Forchielli et al. [36] aimed to compare dietary habits in a vast group of 205 children and adolescents with a long-term adherence to a GFD with Italian dietary recommendations (LARN). Children with CD consumed significantly less energy than LARN recommendations, with particularly low intakes among adolescent females (−25% than recommended). Excessive protein consumption was observed across all age groups, with intake reaching up to 3.5 times the recommended amount for children aged 3–7. Although excessive protein intake remained above the recommended levels, it did slightly decline with age. No significant differences were observed in carbohydrate, lipid, or fiber intake after 12 months on a GFD compared to LARN recommendations.

In a study by Larretxi et al. [39], DRI for the Spanish population and a simulated gluten-containing diet made by replacing gluten-free products with gluten-containing analogs were compared to the distribution of energy and macronutrients in the diet of 83 children and adolescents with CD following a GFD for more than 12 months. Regarding energy intake, no significant difference was found between a GFD and a gluten-containing diet. Nevertheless, energy intake was below the estimated energy expenditure, reaching approximately 80% of the recommended levels. Protein consumption was lower in those on the GFD than on the GCD, although it still accounted for about 17% of total calories. Carbohydrate intake accounted for 45.4% of total energy, falling below the recommendations of the European Food Safety Authority (EFSA). The GFD’s fat consumption was higher than the EFSA’s, accounting for 39.6% of total calories. In addition, compared to the gluten-containing diet, the GFD had lower levels of MUFA and PUFA and higher levels of SFA.

In a study conducted by Ferrara et al. [35], 100 patients, including 50 children with CD adherent to a GFD and a control group of 50 healthy children, were enrolled. A statistically significant increase in total daily fat intake was observed in the CD group compared to the control group, primarily driven by fat consumption from snacks, while mean energy intake showed no significant difference between the two groups.

Similar findings were revealed by another research group [40] regarding energy and fat intake, further extending the analysis to children and adolescents adhering to a GFD for a longer duration of 2 years. Lionetti et al. [40] enrolled 100 children with CD and a control group of 100 healthy children. In contrast to the previous study, the estimated daily energy and protein intake were similar in the two groups, and both protein intake and daily energy intake derived from protein aligned with the LARN recommendations. Although the percentage of energy derived from carbohydrates was the same between groups, reaching the nutritional goal recommended by LARN, the daily intake of carbohydrates and fiber was significantly lower in children with CD. Intakes of total and saturated fats were higher in the CD group compared to HCs, exceeding the LARN’s recommended dietary target of less than 10% of total energy.

Similarly, a study by Szaflarska-Popławska et al. [45] enrolled 48 children with CD. Energy and macronutrient intake were compared to those of 50 non-celiac subjects and the age-specific Polish Dietary Reference Values recommended by the National Institute of Public Health-National Institute of Hygiene (NIPH-NIH) in Warsaw, Poland. Energy, lipid, and fiber intake were similar between children with CD and HC, aligning with the DRIs. However, 47.6% children in both groups consumed fewer calories than recommended, while 42.8% consumed lower amount of lipids and 33.3% consumed lower amounts of fiber. When compared to participants with no CD, the CD group’s protein and carbohydrate intakes were both higher than dietary recommendations, coming in at 190.3% and 189.4% of the DRI, respectively.

In another study by Balamtekin et al. [34], 28 children with CD who had been on a GFD for at least 12 months were included. The control group consisted of 25 age- and gender-matched healthy children. Participants with CD had a significantly lower daily energy, protein carbohydrate, and fiber intake compared to HCs. Although no significant differences were observed in total fat intake between groups, the percentage of daily caloric intake from fat and PUFA was significantly higher in children with CD compared to the HCs.

Ballestero Fernández et al. [22] conducted a cross-sectional age and gender-matched study in 70 children with CD following a GFD for more than 12 months and 67 children without CD. The results show that energy derived from carbohydrates in the diet of children and adolescents with CD was similar to that of controls, but remained below the acceptable macronutrient distribution range for the Spanish population, suggesting that carbohydrates should provide 50–60% of total energy. Regarding protein, while the intake of participants with CD was significantly lower than that of controls, it was still aligned with the recommended levels of 15% of total energy intake. No differences were detected for total lipid and fiber intake between patients and controls. However, lipid contribution to energy intake was high in both groups and dietary fiber intake was low compared to recommendations for the Spanish population.

Rujner et al. [43] compared 41 children with CD following a GFD for a mean duration of 11 years, 28 children with untreated CD, and 8 HCs. Amongst GFD-treated children, energy intake as a percentage of the recommended dietary intake was 139.2% compared to 150.5% in the control group. However, 17% of the treated children consumed less energy than the recommended daily intake (RDI), while none of the HCs fell below the recommended levels. On average, both groups consumed enough protein—113% of the RDI for participants with CD and 141.2% for HCs—and exceeded the RDI for fat—163% and 153.8% of the RDI for patients with CD and HCs, respectively. However, 46% of patients with CD failed to meet protein recommendations compared to the 12.5% of HCs, while 15% of patients with CD consumed insufficient fat, with no deficiencies observed in the HCs.

Sila et al. [44] aiming to assess the quality of the diets of patients with CD in comparison to the HCs, studied 76 patients with CD following a GFD in the long term and 590 HCs. Although calorie intake was higher in children with CD than HCs, they achieved a greater percentage of their estimated energy requirements (EER)–96.4% of their EER compared to 72.7% in HCs. Additionally, children and adolescents with CD achieved a higher percentage of protein and fat requirements compared to HCs as soon as they consumed significantly more protein and fat. In terms of fat, MUFA intake was lower in patients with CD, while no significant differences were observed for PUFA and SFA intake. Inadequate amount of fiber was evident in both groups.

Currently, three prospective studies have been published investigating changes in diet during the first year following a GFD in newly diagnosed children and adolescents [37,38,41].

Specifically, in a study by Forchielli et al. [37] researchers prospectively analyzed dietary habits and their consistency with LARN recommendations in children and adolescents with CD at the time of diagnosis and during the first year on a GFD. Caloric intake decreased marginally during the year on a GFD, while, by the end of the first year, it was 13% less than LARN recommendations. Protein intake remained 200% more than recommended. From baseline until one year on a GFD, fat intake as a proportion of total energy intake stayed at 34%, which is in line with recommendations. Saturated fat intake dropped from 12.7% to 11.2% of total energy intake, with cold cuts and dairy products being the main sources of saturated fat. Unsaturated fat intake went from 18.6% to 19.2, while, over the course of the year, the omega-6 to omega-3 ratio improved, going from 13.3 ± 5.5 to 8.8 ± 3.1, which is below the often seen 10:1 ratio in Western countries. After excluding gluten, consumption of carbohydrates dropped from 54% of total daily energy intake to 52%, even though fiber intake increased and almost reached the recommended level.

In contrast to the previous reported study, Kozioł-Kozakowska et al. [38] showed that the amount of energy consumed increased by 110.70 kcal at 6 months on a GFD and 144.54 kcal at 12 months on a GFD compared to baseline, while no changes were observed for the percentage of implementation EER norm and macronutrients during the year on GFD. Similarly, Mager et al. [41] carried out a longitudinal case series study that aimed to examine the potential interrelationships between nutritional status and bone accrual and BMD at time of diagnosis and after 1 year on a GFD in 22 children and adolescents with CD. At the end of the first year, energy and protein intake decreased by 202 kcal and 4.8 g, respectively. In terms of protein, although total intake decreased, it remained nearly double the Recommended Dietary Allowance (RDA).

Overall, the studies evaluating short-term and long-term nutrient intake in children and adolescents with CD reveal significant variability in energy and macronutrient intake, with results differing across studies depending on the comparison tool used (i.e., HCs, DRI, RDA, EFSA recommendations, time of diagnosis). Nevertheless, some consistent findings can be identified across the studies. Findings from the short-term cross-sectional and case–control studies indicate that children and adolescents with CD consume higher amounts of protein and carbohydrate compared to HCs. Over the long term, fat intake increased, while intake of protein remained excessive compared to HCs, with both macronutrients frequently surpassing dietary recommendations. In contrast, fiber intake was similar between patients with CD and HCs; however, in both groups, it was often below the recommended levels. Current evidence from prospective studies examining dietary changes during the first year on a GFD are not clear.

### 3.4. Associations of GFD with Micronutrient Intake

Methodological diversity was also noted across all studies that assessed micronutrient intake regarding the comparison group that they used (i.e., DRI, HCs, gluten-containing diet, and baseline). To provide a more comprehensive evaluation of micronutrient status, the present systematic review also evaluated the blood micronutrient status reported in five studies [22,33,38,41,45]. However, none of the studies included a blood analysis for trace minerals.

#### 3.4.1. Fat-Soluble Vitamins

The dietary intake of fat-soluble vitamins has been examined within the context of a GFD. A special focus was given on Vitamin D due to its association with bone health and the increased risk of reduced bone mineral density (BMD) observed in children with CD with vitamin D deficiency compared to the general population [47,48]. The main results of the studies are shown in Table 3.

Specifically, observational studies evaluating fat-soluble vitamin intake in children and adolescents with CD following a GFD in the short term are lacking. Allowaymi et al. [33] showed that dietary intake of Vitamin A, D, and E were significantly lower than the DRI, while with regard to vitamin K dietary intake, boys exhibited lower intake compared to the recommended values. With regard to blood levels, vitamin D levels were lower than the reference range for both genders. Additionally, Zuccotti et al. [46] found inadequate dietary intake of Vitamin D in both patients and HCs. Of note, mean intake of vitamin D was found to be lower in children with CD compared to HCs.

With regard to the long-term adherence to a GFD, data from epidemiological studies generally support the idea that a GFD is associated with adequate intake of Vitamin D, whereas mixed results are shown for the rest of fat-soluble vitamins.

Specifically, Larretxi et al. [39] indicated higher levels of vitamin E but lower levels of Vitamin A and Vitamin D on a GFD compared to a simulated gluten-containing diet. Three research groups [22,44,45] reached similar conclusions regarding Vitamin D with inadequate dietary intake for both participants with CD and HCs, highlighting an overall dietary inadequacy rather than CD-specific factors. Additionally, Ballestero-Fernandez et al. [22] found that Vitamin A and Vitamin K dietary intake exceeded the recommended levels, while Vitamin E consumption was found to be insufficient across all groups. In contrast, Balamtekin et al. [34] found that the 28 children with CD consumed enough vitamin E, which was significantly higher with respect to the HCs. There were no between-groups significant differences regarding Vitamin A intake.

Kozioł-Kozakowska et al. [38], through a prospective cohort study, evaluated the dietary intake of CD children with CD in three time points: before diagnosis, after six months, and following one year of GFD. Adherence to the nutritional recommendations remained consistently low for vitamins E and K across all time points, while vitamin A intake met recommendations. Although it was still far below the required levels, vitamin D intake did show a statistically significant improvement over time, rising from the point of diagnosis to the short term and further improving from six months to the long-term adherence to a GFD. Along similar lines, Mager et al. [41] found that, at time of diagnosis, 90% of the participants did not achieve the estimated average requirement for vitamin D, with none meeting them after one year on a GFD, partly due to poor sunlight exposure which can lead to reductions in the endogenous synthesis of vitamin D. Additionally, regarding vitamin K, 40.9% and 31% of the participants had a dietary intake below 50% of the adequate intake at the time of diagnosis and in a long-term gluten-free diet, respectively.

**Table 3:** Summary of the findings on the association between GFD and fat-soluble vitamin intake.

Study	Duration of GFD	Results			
		Vitamin A	Vitamin D	Vitamin E	Vitamin K
Allowaymi et al. [33]	Short term ≥6 months	↓ (compared to DRI) G: 247.09 μg (*p* = 0.000) B: 326.16 μg (*p* = 0.004) DRI: 600 μg	↓ (compared to DRI) G: 2.50 mg (*p* = 0.000) B: 3.26 mg (*p* = 0.000) DRI: 15 mg	↓ (compared to DRI) G: 1.59 mg (*p* = 0.000) B: 1.45 mg (*p* = 0.000) DRI: 11 mg	↓ for boys (compared to DRI) NS for girls G: 62.25 μg (*p* = 0.865) B: 34.71 μg (*p* = 0.021) DRI: 60 μg
Zuccotti et al. [46]	Short term ≥6 months	-	↓ (*p* < 0.001) (compared to HCs) CD: 0.8 ± 0.6 μg HC: 3.1 ± 2.8 μg	-	-
Ballestero- Fernández et al., 2019 [22]	Long term ≥1 y	NS	NS	NS	NS
Balamtekin et al., 2015 [34]	Long term 4.0 ± 3.3 y	NS	-	↑ (*p* = 0.004) (compared to HCs) CD: 19.7 ± 6.5 mgHC: 14.3 ± 6.5 mg	-
Larretxi et al., 2018 [39]	Long term 6.43 ± 4.18 y	↓ (*p* < 0.001) (compared to GCD) GF: 555 ± 224 μg GC: 602 ± 231 μg	↓ (*p* < 0.001) (compared to GCD) GF: 3.3 ± 3.5 μgGC: 3.8 ± 3.3 μg	↑ (*p* < 0.001) (compared to GCD) GF: 10.4 ± 3.9 mgGC: 9.6 ± 3.4 mg	-
Sila et al., 2020 [44]	Long term 34.1 ± 25.4 y	-	↑ (*p* < 0.001) (compared to HCs) CD: 2.2 ± 2.4 μgHC: 1.1 ± 1.7 μg	-	-
Szaflarska-Popławska et al., 2022 [45]	Long term 5.02 ± 3.87 y	-	NS	-	-
Kozioł-Kozakowska et al., 2021 [38]	Diagnosis (T0) Short term 6 months (T1) Long term 12 months (T2)	NS	↓Before diagnosis ↑ (*p* < 0.01) After 6mo and 12 months T0: 1.72 ± 0.92 μg 11.47 ± 6.19% RDA T1: 2.15 ± 1.74 μg 14.30 ± 11.58% RDA T2: 3.59 ± 8.09 μg 23.90 ± 53.91% RDA	↓ Before diagnosis 6.15 ± 2.98 μg 87.01 ± 41.68% RDA NS after 6mo and 12 months	↓ Before diagnosis 4.88 ± 7.59 μg 8.43 ± 13.50% RDA NS after 6mo and 12 months
Mager et al., 2011 [41]	Diagnosis (T0) Long term 12 months (T2)	NS	↓ (*p* < 0.05) (compared to time of diagnosis) T0:4.7 ± 4.3 μg 31.1 ± 28.8% RDA T2: 3.2 ± 2.3 μg 21.1 ± 15.1% RDA	-	NS after 12 months (compared to time of diagnosis)

Overall, vitamin D intake and plasma levels were consistently inadequate in both short-term and long-term adherence to a GFD and were frequently well below recommended levels. It is noteworthy that this deficiency was observed not only in participants with CD, but also in HC participants, suggesting a generally inadequate diet rather than CD-specific causes. Although vitamin D intake improved slightly over time in some studies, it generally remained far from the required levels. Studies focusing on vitamins A, E, and K are limited. Nevertheless, there is evidence that children with CD may be at risk of vitamin E and K deficiency. In contrast to other fat-soluble vitamins, vitamin A appears to be less of a concern, as children and adolescents with CD who adhere to a GFD are generally less likely to be deficient in this particular vitamin than children with HC.

#### 3.4.2. Water Soluble Vitamins

To date, studies in the adult population consistently report low intakes of B-complex vitamins, particularly thiamin (Vitamin B1), riboflavin (Vitamin B2), and pyridoxine (Vitamin B6) [20]. The relationship between Vitamin B12 and folate levels in patients with CD on a GFD remains unclear. While some researchers support the idea that these nutrient deficiencies appear to be generally corrected in patients with CD adhering to a GFD [49], others report a high prevalence of deficiency [50,51].

Furthermore, data from the adult population suggest that these deficits may contribute, at least in part, to the increased prevalence of cardiovascular disease (CVD) in people with CD. One contributing factor could be the nutritional inadequacy of gluten-free products, which contain significantly fewer essential nutrients such as vitamin B12 and folic acid compared to their gluten-containing counterparts [52]. Similar concerns apply to the pediatric population with CD, as intake of B-complex vitamins has been reported to be inadequate [17]. Table 4 summarizes the findings concerning dietary consumption of water-soluble vitamins.

In the short-term adherence to a GFD, Allowaymi et al. [33] observed that children with CD consumed thiamin, pyridoxine, and folate below the DRI. Additionally, girls failed to meet the recommendations for riboflavin, niacin, and Vitamin C. No significant difference was found for Vitamin B12. In a similar vein, Nestares et al. [42] and Zuccotti et al. [46] did not reveal differences in water-soluble vitamin intake between children with CD and HCs.

Regarding the long-term adherence to a GFD, Larretxi et al. [39] noted that a small percentage of participants with CD had inadequate intakes of thiamine, riboflavin, niacin, vitamin B6 and B12, while more than half of the participants did not reach the recommendations for optimal intake of folic acid. When the researchers compared the GFD with a simulated GCD, they also found lower levels of thiamine, riboflavin, niacin, vitamin B6, vitamin B12, folic acid and biotin.

In terms of case–control studies, Ballestero Fernández et al. [22] revealed that children and adolescents with CD had lower thiamine, niacin, Vitamin B6, and folate intake with respect to HCs. Nevertheless, thiamin, pyridoxine, and niacin intake were well above the recommendations, and absolute intake may be considered adequate, while folate intake was insufficient, especially for girls and children. In contrast, plasma folate concentrations were within normal levels, with no differences observed between children and adolescents with or without CD. Vitamin B12 intake exceeded recommendations in both groups.

Additionally, Balamtekin et al. [34] noted lower thiamine intake from children and adolescents with CD compared to HCs. In contrast, in a study by Szaflarska-Popławska et al. [45], the mean daily intake of thiamine, riboflavin, niacin, Vitamin B6, and B12 in children with CD was above the dietary recommendations, while the mean intake of folic acid was low. These figures, however, were not significantly different from the control group’s mean intake. Biochemical analysis showed that 14.6% of children with CD had thiamine and folic acid deficit (mean serum levels of 62.8 ± 12.3 mg/L and 7.9 ± 2.9 ng/mL, respectively) with values being significantly lower compared to the HCs. A similar prevalence of serum vitamin B2 deficiency was noted in both groups.

As far as Vitamin C intake is concerned, three study groups, Ballestero Fernández et al. [22], Sila et al. [44], and Szaflarska-Popławska et al. [45], found that children and adolescents with CD exceeded the median percentage of the recommended intake, while Sila et al. [44] reported that children with CD on a GFD were more compliant with the nutritional norms for Vitamin C compared to HCs.

Two prospective studies investigated changes in the diet of children and adolescents with CD from the time of diagnosis to long-term GFD. Specifically, Kozioł-Kozakowska et al. [38] showed that there is no risk of deficiency in water-soluble vitamins in the short or long term, with the exception of folic acid. The implementation of the dietary norm for folic acid appeared to be low at the beginning of the study, while after one year of adherence to the GFD, adherence to dietary requirements remained unchanged due to the low consumption of vegetables, especially green vegetables. On the other hand, vitamin C intake increased significantly in both the short and long term. Likewise, in a study conducted by Forchielli et al. [37] folate dietary intake decreased over the year on a GFD. When participants were grouped by age (1–9, 10–14 and 15–18 years), patients in the youngest cohort had lower folic acid levels overall, with the decrease being more pronounced in females than in males.

Overall, there are limited data on the role of GFD in the short-term status of water-soluble vitamins. The results suggest that children and adolescents with CD have lower intakes of vitamins B1, B2, B3, B6, and folic acid compared to DRI. In the long term, children and adolescents on a GFD are at high risk of vitamin B1, B2, B3, B6, and folic acid deficiency. Remarkably, these values did not differ from those of the HCs. In contrast, the intake of vitamin B12 and vitamin C generally met or exceeded the recommendations. Prospective studies have shown that folic acid intake did not improve in the first year of GFD, while vitamin C intake increased in the long term.

#### 3.4.3. Essential Minerals

Specific deficiencies in essential minerals, particularly iron and calcium, associated accordingly with anemia and osteoporosis, are more prevalent among people with CD [20]. A summary of the studies that have reported results with regard to the dietary intake of essential minerals is included in Table 5.

In the short term, Allowaymi et al. [33] indicated that children and adolescents with CD consumed less calcium, magnesium, and potassium compared to the DRI, whereas girls did not reach the recommended levels for iron. In addition, children and adolescents with CD adhered to the dietary norms for sodium. In terms of blood levels, boys with CD had higher potassium levels than girls, while calcium levels were within the normal range for both genders. Nestares et al. [42] found that iron and magnesium intake was lower for children with CD on a GFD in respect to the control group, with no significant difference in calcium intake. In a study conducted by Zuccotti et al. [46], while no differences were observed in the iron, magnesium, and calcium dietary intake between children and adolescents with CD and HCs, both groups did not align with the recommendations for iron and calcium intake.

**Table 4:** Summary of the findings on the association between GFD and water-soluble vitamin intake.

Study	Duration of GFD	Results						
		Vitamin B1	Vitamin B2	Vitamin B3	Vitamin B6	Vitamin B12	Folate	Vitamin C
Allowaymi et al., 2022 [33]	Short term ≥6 months	↓ (compared to DRI) G: 0.53 mg (*p* =0.000) B: 0.69 mg (*p* =0.022) DRI:0.9 mg	↓ for girls (compared to DRI) NS for boys G: 0.72 mg (*p* = 0.004) B: 0.94 mg (*p* = 0.683) DRI: 0.9 mg	↓ for girls (compared to DRI) NS for boys G: 7.97 mg (*p* = 0.000) B: 9.62 mg (*p* = 0.062) DRI: 12 mg	↓ (compared to DRI) G: 0.54 mg (*p* = 0.000) B: 0.66 mg (*p* = 0.000) DRI: 1.0 mg	NS for boys	↓ (compared to DRI) G:146.52 μg (*p* = 0.000) B:142.09 μg (*p* = 0.000) DRI: 300 μg	↓ for girls (compared to DRI) NS for boys G: 32.24 mg (*p* = 0.003) B: 36.18 mg (*p* = 0.104) DRI: 45 mg
Zuccotti et al., 2012 [46]	Short term ≥6 months	-	-	-	NS	NS	NS	-
Nestares et al., 2020 [42]	Short term >6 months	-	-	-	-	NS	NS	-
Ballestero- Fernández et al., 2019 [22]	Long term ≥1 y	↓ (*p* < 0.05) (compared to HC) CD: 110.0% of recommendations HC: 133.3% of recommendations	↓ (*p* < 0.05) (compared to HC) CD: 107.1% of recommendations HC: 121.4% of recommendations	↓ (*p* < 0.05) (compared to HC) CD: 177.4% of recommendations HC: 231.4% of recommendations	↓ (*p* < 0.05) (compared to HC) CD: 118.5% of recommendations HC: 141.9% of recommendations	NS	↓ (*p* < 0.05) (compared to HC) CD: 67.45% of recommendations HC: 82.0% of recommendations	NS
Balamtekin et al., 2015 [34]	Long term 4.0 ± 3.3y	↓ (*p* < 0.001)(compared to HC) CD: 0.6 ± 0.1 mgHC: 0.8 ± 0.1 mg	NS	-	NS	-	NS	NS
Larretxi et al., 2018 [39]	Long term 6.43 ± 4.18 y	↓ (*p* < 0.001) (compared to GCD) GF: 1.3 ± 0.4 mgGC: 1.4 ± 0.5 mg	↓ (*p* < 0.001) (compared to GCD) GF: 1.6 ± 0.4 mgGC: 1.8 ± 0.5 mg	↓ (*p* < 0.001) (compared to GCD) GF: 21.2 ± 5.6 mgGC: 23.7 ± 6.2 mg	↓ (*p* < 0.001) (compared to GCD) GF: 1.6 ± 0.4 mgGC: 1.9 ± 0.5 mg	↓ (*p* < 0.001) (compared to GCD) GF: 5.9 ± 2.9 μgGC: 6.8 ± 3.7 μg	↓ (*p* < 0.001) (compared to GCD) GF: 186 ± 76 μgGC: 233 ± 83 μg	NS
Sila et al., 2020 [44]	Long term 34.1 ± 25.4 y	NS	-	-	-	-	-	↑ (*p* = 0.004)(compared to HC) CD: 91.9 ± 51.8 mgHC: 74.1 ± 49.8 mg
Szaflarska-Popławska et al., 2022 [45]	Long term 5.02 ± 3.87 y	↓ (*p* < 0.001) (compared to HC) CD:0.6 ± 0.1 mgHC:0.8 ± 0.1 mg	NS	NS	NS	NS	NS	NS
Forchielli et al., 2020 [37]	Diagnosis (T0) Short term 6 months (T1) Long term 12 months (T2)	-	-	-	-	-	↓ (*p* < 0.001) (after 12 months) T0: 155.5 ± 66.6 μg T2: 118.4 ± 63.5 μg	-
Kozioł-Kozakowska et al., 2021 [36]	Diagnosis (T0) Short term 6 months (T1) Long term 12 months (T2)	NS	NS	NS	NS	NS	↓ Before diagnosis 160.96 ± 60.03 μg 58.20 ± 24.26% RDA NS after 6 months and 12 months	↑ (*p* <0.01) After 6 and 12 months Before diagnosis: 63.92 ± 36.48 mg 123.80 ± 75.58% RDA T1: 81.70 ± 53.98 mg 156.77 ± 102.89% RDA T2: 93.21 ± 55.10 mg 179.73 ± 122.25% RDA

In the long term, Larretxi et al. [39] observed that a GFD is lower in iron, magnesium, and sodium, higher in calcium and potassium, and comparable to a GCD in phosphorus. Furthermore, nearly 10% of the participants with CD showed insufficient intakes of magnesium, 25% had inadequate iron intake, and more than the half did not fulfill the recommended calcium intake. Along similar lines, Forchielli et al. [36] found that calcium and iron intakes were below the LARN recommendations, while sodium intake exceeded the recommended levels.

Concerning case–control studies, evidence from three research groups [22,34,45] suggested that patients with CD may be at a higher risk of iron deficiency, with Szaflarska-Popławska et al. [45] highlighting that all participants failed to meet the recommended levels regardless of whether they were following a GFD. Furthermore, magnesium intake showed mixed results, with one study [45] reporting levels exceeding the recommendations and two studies [22,34] identifying participants with CD at risk of deficiency. However, Rujner et al. [43] found that the average magnesium intake was similar between children and adolescents with CD and HCs, aligning with Polish recommendations. Calcium intake was also different across studies. Sila et al. [44] found that participants with CD had higher calcium dietary intake, while Ballestero Fernández et al. [22] found lower intake, and other studies found no significant differences between the CD and control groups [34,45]. Interestingly, Szaflarska-Popławska et al. [45] noticed that both groups had insufficient calcium intake.

In terms of mineral blood levels, Szaflarska-Popławska et al. [45] noticed that although serum calcium levels were within normal ranges, they were significantly higher in the control group than in the CD group. Additionally, Ballestero Fernández et al. [22] observed that despite the reduced dietary intakes of iron, folate, and calcium, blood concentrations of these nutrients were within normal range and there were no differences between children and adolescents with CD and HCs.

In a prospective study by Kozioł-Kozakowska et al. [36], the researchers highlighted that the inadequate dietary iron intake, as well as the low serum iron levels, are challenging, especially since there was no significant increase in iron dietary intake, nor, consequently, in blood iron levels during the year. However, potassium dietary intake showed improvement from the time of diagnosis to both the short term and long term. In another prospective study by Mager et al. [41], the intake of calcium and sodium was lower after long-term adherence to a GFD compared to baseline. In both studies [38,41], the intake of phosphorus and magnesium remained stable over the year.

Overall, the intake of calcium, magnesium, phosphorus, and potassium is below the DRI in the short term, while the intake of iron and magnesium is lower in children and adolescents with CD compared to HCs. In the long term, potassium intake improves, but sodium intake remains inadequate in most studies. In addition, the intake of iron, calcium, and magnesium is inadequate not only in children with CD, but also in healthy peers, as some studies show. Prospective studies examining nutritional status in relation to essential minerals after one year of GFD show that calcium and iron intake was inadequate from the baseline. These deficiencies persist in both the short and long term without any significant improvement. However, promising trends can be observed in the restoration of potassium levels.

**Table 5:** Summary of the findings on the association between GFD and essential mineral intake.

Study	Duration of GFD	Results					
		Calcium	Iron	Magnesium	Phosphorus	Sodium	Potassium
Allowaymi et al., 2022 [33]	Short term ≥6 months	↓ (compared to DRI) G: 496.58 mg (*p* < 0.001) B: 531.45 mg (*p* < 0.001) DRI: 1300 mg	↓for girls (compared to DRI) No significant difference for boys G: 6.22 mg (*p* < 0.001) B: 7.68 mg (*p* = 0.666) DRI: 8 mg	↓ (compared to DRI) G: 94.64 mg (*p* < 0.001) B: 97.85 mg (*p* < 0.001) DRI: 240 mg	↓ (compared to DRI) G: 458.84 mg (*p* < 0.001) B: 494.23 mg (*p* < 0.001) DRI: 1250 mg	NS	↓ (compared to DRI) G: 1032.87 mg (*p* < 0.001) B: 1175.18 mg (*p* < 0.001) DRI: 2500 mg
Zuccotti et al., 2012 [46]	Short term ≥6 months	NS	NS	NS	NS	NS	NS
Nestares et al., 2020 [42]	Short term >6 months	NS	↓ (*p* = 0.006) (compared to HC) CD: 7.61 ± 0.49 mg HC: 10.1 ± 0.58 mg	↓ (*p* < 0.001) (compared to HC) CD: 165.6 ± 9.23 mg HC: 206.9 ± 10.9 mg	-	-	-
Ballestero- Fernández et al., 2019 [22]	Long term ≥1 y	↓ (*p* < 0.05) CD: 64.1% of recommendations HC: 74.7% of recommendations	↓ (*p* < 0.05) CD: 76.0% of recommendations HC: 103.1% of recommendations	↓ (*p* < 0.05) CD: 74.4% of recommendations HC: 82.8% of recommendations	NS	-	-
Balamtekin et al., 2015 [34]	Long term 4.0 ± 3.3y	NS	↓ (*p* < 0.001)(compared to HCs) CD: 6.9 ± 2.6 mgHC: 11.2 ± 3.2 mg	↓ (*p* = 0.01)(compared to HCs) CD: 200.5 ± 68.3 mgHC: 247.6 ± 65.3 mg	↓ (*p* = 0.005)(compared to HCs) CD: 899.6 ± 247.7 mgHC: 1088.8 ± 223.8 mg	↓ (*p* < 0.001)(compared to HCs) CD:972.7 ± 363.3 mgHC:1655.5 ± 822.8 mg	NS
Forchielli et al., 2015 [36]	Long term 6.2 ± 4.1 y	↓ (*p* ≤ 0.001) (compared to LARN) CD:595.4 ± 305 mg LARN:1091.1 ± 295.3 mg	↓ (*p* < 0.0001)(compared to LARN) CD:6.9 ± 2.6 mgLARN:11.2 ± 3.2 mg	-	-	↑ (*p* = 0.002) (compared to LARN) CD:1529.3 ± 1317.8 mgLARN:1173.6 ± 353.7 mg	-
Larretxi et al., 2018 [39]	Long term 6.43 ± 4.18 y	↑ (*p* = 0.007) (compared to GCD) GF: 900 ± 217 mgGC: 887 ± 308 mg	↓ (*p* < 0.001) (compared to GCD) GF: 12.2 ± 3.3 mgGC: 13.3 ± 3.4 mg	↓ (*p* < 0.001) (compared to GCD) GF: 242 ± 64 mgGC: 279 ± 69 mg	NS	↓ (*p* < 0.001) (compared to GCD) GF: 1819 ± 529 mgGC: 1932 ± 533 mg	↑ (*p* = 0.001) (compared to GCD) GF: 2788 ± 585 mgGC: 2747 ± 555 mg
Sila et al., 2020 [44]	Long term 34.1 ± 25.4 y	↑ (*p* < 0.001) (compared to HCs) CD: 713.3 ± 261.2 mgHCs: 579.9 ± 242.1 mg	NS	-	-	-	-
Szaflarska-Popławska et al., 2022 [45]	Long term 5.02 ± 3.87 y	NS	NS	NS	-	-	-
Rujner et al. [43]	Long term 2.7–17.3 y	-	-	NS	-	-	-
Mager et al., 2011 [41]	Diagnosis (T0) Long term 12 months (T2)	↓ (*p* < 0.05) after 12 months (compared to time of diagnosis) T0: 994 ± 697 mg 88.8 ± 59.9% RDA T2: 839 ± 349 mg 70.8 ± 31.7% RDA	-	NS	NS	↓ (*p* < 0.05) (after 12 months) (compared to time of diagnosis) T0: 2752 ± 1396 mg 160.4 ± 53.8% RDA T2: 2104 ± 600 mg 123.8 ± 68.3% RDA	-
Kozioł-Kozakowska et al., 2021 [36]	Diagnosis (T0) Short term 6 months (T1) Long-term 12 months (T2)	↓Before diagnosis 537.13 ± 233.32 mg 50.56 ± 23.60% RDA No significant difference after 6 months and 12 months	↓Before diagnosis 8.40 ± 3.01 mg 79.29 ± 38.82% RDA No significant difference after 6 months and 12 months	NS	NS	NS	↑ (*p* <0.03) After 6 months and 12 months T0: 2055.26 ± 687.85 mg 119.68 ± 6.19% RDA T1: 2901.67 ± 685.63 mg 128.51 ± 56.75% RDA T2: 2335.84 ± 568.94 mg 137.88 ± 60.32% RDA

#### 3.4.4. Trace Minerals

Nine studies (eight cross-sectional/case–control and one prospective) examined nutrient intake in terms of trace minerals in children and adolescents with CD on a GFD. Results regarding zinc intake on a GFD are conflicting and not conclusive, while few studies have been constructed on selenium, iodine, chlorine, copper, and chromium. Relevant information about essential minerals dietary intake is included in Table 6.

Allowaymi et al. [33] reported that, in the short-term adherence to a GFD, children and adolescents with CD failed to meet the DRI for zinc. In another study by Nestares et al. [42], researchers found that selenium intake was lower in children with CD compared to HCs, although zinc and iodine intake did not differ significantly between the two groups.

With regard to the adherence to a GFD in the long term, Balamtekin et al. [34] found that zinc intake was significantly lower in patients with CD compared to controls. On the other hand, Szaflarska-Popławska et al. [45] showed that the mean daily intake of zinc both in children with CD and HCs was above the DRI, with 57.2% of children with CD exceeding the recommended levels. However, 33.3% of them had an insufficient zinc intake. Additionally, Larretxi et al. [39] highlighted the potential positive impact of the GFD in terms of the zinc content, noting that it contains twice the amount of zinc compared to a simulated GCD. However, the findings for other trace minerals were contradictory, with the results showing that their levels were lower in a GFD compared to a GCD.

Two research groups [44,46] did not find any significant difference in zinc intake when comparing patients with healthy children and adolescents. Additionally, in a study conducted by Ballestero Fernández et al. [22], zinc and iodine intakes were found to be borderline adequate, with no significant differences between participants with CD and HCs. Children with CD consumed less selenium than HCs. Nevertheless, it significantly exceeded the recommendations, indicating that overall intake was sufficient.

In a prospective study, Kozioł-Kozakowska et al. [36] found that adherence to dietary guidelines for iodine and selenium was notably poor. While adherence to a GFD was not associated with improvement of selenium intake over time, the percentage of the individuals meeting the RDA for zinc and iodine increased both in the short term and long term.

Overall, the relationship between trace elements and GFD in children and adolescents with CD is not clear, both in the short and long term. The currently limited data depend on studies with methodological differences that may lead to conflicting conclusions. In general, selenium intake appears to be consistently lower in children and adolescents with CD compared to healthy peers. The results of zinc intake in patients are contradictory. Some studies suggest a deficiency, while others report adequate intake. Finally, there are limited data on other trace elements, so further research is needed.

**Table 6:** Summary of the findings on the association between GFD and trace mineral intake.

Study	Duration of GFD	Results					
		Zinc	Selenium	Iodine	Chlorine	Copper	Chromium
Allowaymi et al., 2022 [33]	Short term ≥6 months	↓ (compared to DRI) G: 3.15 mg (*p* = 0.000) B: 3.37 mg (*p* = 0.000) DRI: 8 mg	-	-	-	-	-
Zuccotti et al., 2012 [46]	Short term ≥6 months	NS	-	-	-	-	-
Nestares et al., 2020 [42]	Short term >6 months	NS	↓ (*p* < 0.001) (compared to HC) CD: 45.5 ± 3.5 μg HC: 68.9 ± 4.2 μg	NS	-	-	-
Ballestero- Fernández et al., 2019 [22]	Long term ≥1 y	NS	↓ (*p* < 0.05) CD: 159.9% of recommendations HC: 268.3% of recommendations	NS	NS	-	-
Balamtekin et al., 2015 [34]	Long term 4.0 ± 3.3 y	↓ (*p* < 0.001)(compared to HC) CD: 5.9 ± 1.8 mgHC: 9.2 ± 2.1 mg	-	-	-	-	-
Larretxi et al., 2018 [39]	Long term 6.43 ± 4.18 y	↑ (*p* < 0.001) (compared to gluten-containing diet) GF: 15.1 ± 13.3 mgGC: 8.7 ± 2.6 mg	↓ (*p* < 0.001) (compared to gluten-containing diet) GF: 36.9 ± 17.6 μgGC: 52.0 ± 20.2 μg	↓ (*p* < 0.001) (compared to gluten-containing diet) GF: 63.0 ± 28.6 μgGC: 75.3 ± 32.1 μg	↓ (*p* < 0.001) (compared to gluten-containing diet) GF: 697 ± 322 mgGC: 1118 ± 518 mg	↓ (*p* < 0.001) (compared to gluten-containing diet) GF: 0.9 ± 0.4 mgGC: 1.2 ± 0.5 mg	-
Sila et al., 2020 [44]	Long term 34.1 ± 25.4 y	NS	-	-	-	-	-
Szaflarska-Popławska et al., 2022 [45]	Long term 5.02 ± 3.87 y	NS	-	-	-	-	-
Kozioł-Kozakowska et al., 2021 [36]	Diagnosis (T0) Short term 6 months (T1) Long term 12 months (T2)	↑ (*p* <0.01) After 6mo and 12 months T0: 6.5 ± 0.92 mg 114.08 ± 44.97% RDA T1: 6.86 ± 1.75 mg 119.27 ± 35.84% RDA T2: 7.48 ± 1.57 mg 129.51 ± 39.58% RDA	↓ Before diagnosis 6.04 ± 7.62 μg 18.86 ± 25.26% RDA NS after 6 months and 12 months	↓ Before diagnosis ↑ (*p* <0.01) After 6 months and 12 months T0: 13.57 ± 6.74 μg 13.23 ± 7.68% RDA T1: 18.08 ± 8.20 μg 17.38 ± 11.58% RDA T2: 16.53 ± 6.84 μg 15.79 ± 6.90% RDA	-	NS	-

### 3.5. Associations of the GFD with Anthropometric Characteristics

From the 15 studies included in the present systematic review, all of them explored the associations of a GFD with diverse anthropometric measurements. The anthropometric indicators examined for assessing growth and body composition varied across the studies. These included body weight [33,36,37,39,40,41,42,44,46], body height [33,36,37,39,40,41,42,44,46], body fat percentage [33], waist circumference [37], carpal wrist circumference [37], waist-to-height ratio [37], fat mass [33], fat-free mass [33], muscle mass [33], and body mass index (BMI) [22,33,36,37,38,39,40,41,44,46]. As it is known, a transformation into z-scores is required because children’s anthropometric values vary based on age and gender [53]. From the included studies, values of body weight z-score [41,44,45,46], body height z-score [41,44,45,46], and BMI z-score [33,39,41,44,45,46] were used.

Several definitions of underweight, overweight, and obesity were used in the included studies. Three research groups [33,38,45] used standard deviation (SD), defined underweight as BMI <−2 SD, overweight as BMI >+1 SD, obesity as BMI >+2 SD, and short stature as <−2 SD. Kozioł-Kozakowska et al. [38] applied OLAF percentile charts for children older than six years old. Four research groups [22,36,39,41] used national and international growth standards. Ballestero Fernández et al. [22] classified participants BMI cut-off points proposed by both the Fundación Orbegozo (Spain) and the World Health Organization (WHO), while Forchielli et al. [36] applied the Italian growth standards. for their classification, Larretxi et al. [39] used age- and sex-standardized BMI cut-off points derived from both national and international databases to classify thinness and overweight. Finally, Mager et al. [41] applied Tanner staging to evaluate the participants’ growth and pubertal development.

Regarding the assessment of height, all studies consistently reported that children with CD have a shorter stature compared to their healthy peers. In the short term, in a study by Nestares et al. [42], researchers showed differences in height between the children with CD and HCs; the mean height in the 68 participants with CD was 129.2 cm, which was significantly shorter compared to the 48 HC peers, with a mean height of 142.2 cm. Furthermore, Allowaymi et al. [33], aiming to evaluate the nutritional status of children with CD who followed the Ministry of Health’s GFD program, noted that short stature was more prevalent in boys (13.8%) than in girls (2.7%) with CD. Additionally, in the long term, evidence from two research groups who investigated nutritional imbalances in children with CD highlighted the potential negative impact of a GFD on height measures [44,45]. Specifically, when compared to 590 HCs, Sila et al. [44] observed that 76 participants with CD had significantly lower height z-scores compared to controls (mean ± standard deviation (SD)) 0.3 ± 1.1 for the CD group and 1.3 ± 1.1 for the controls). In the study by Szaflarska et al. [45], lower height-for-age z-score was linked to a GFD, since the 48 participants with CD had considerably lower height-for-age z-scores than the 50 healthy peers, with mean values ± SD of–0.06 ± 1.17 for the CD group and 0.57 ± 1.15 for the control group.

As far as weight and BMI are concerned, in the short-term, Allowaymi et al. [33] noted that most children with CD exhibited a normal BMI for their age, with 89.7% of boys and 67.6% of girls categorized as normal. Girls demonstrated marginally elevated prevalence of both overweight and underweight compared to boys, while obesity occurred in only one girl.

Concerning the adherence to a GFD for a long-term, similar results were reported in a study by Ballestero Fernández et al. [22]. Researchers indicated that more than 62% of both patients and controls maintained a normal BMI, while a tendency for increased thinness was noticed in patients with CD and increased overweight and obesity tendency in HC. However, Larretxi et al. [39] found that approximately 70% of children with CD on a long-term GFD had a normal BMI, while 20% were classified as underweight and 11% as overweight, with no cases of obesity reported. Forchielli et al. [36] reported that the mean BMI of the cohort, consisting of 203 individuals, was 18.2 ± 3.2; notably, 7.8% of the children (n = 16) had a BMI beyond the 85th percentile.

Sila et al. [44] highlighted additional long-term effects, noting that patients with CD had significantly lower body weight z-score (0.2 ± 1.0 in patients with CD vs. 0.8 ± 0.9 in HCs) compared to HCs, although BMI z-scores showed no significant difference. Moreover, Szaflarska et al. [45] noted that patients with CD following a GFD over one year had significantly lower weight-for-age z-score and BMI-for-age z-score compared to HCs. A significantly higher number of patients were classified as underweight, while a significantly lower number were classified as overweight or obese compared to the controls.

Zuccotti et al. [30], studying children on a short-term GFD, and Ballestero Fernández et al. [22] and Lionnetti et al. [31], focusing on children on a long-term GFD, reported no association between a long-term adherence to a GFD and anthropometric measurements. Furthermore, Zuccotti et al. [46] observed that both children with and HCs had BMI values aligned with Italian BMI guidelines, indicating similar growth patterns.

Four studies evaluated the anthropometric changes after one year on a GFD in newly diagnosed children with CD [36,38,41,44]. Specifically, in a study by Kozioł-Kozakowska et al. [38], even though body height and weight increased by an average of 8 cm and 4 kg during the course of the year, BMI or BMI percentile did not show any significant results. Furthermore, at the time of diagnosis, 67.5% of children had a normal BMI, 20% were underweight, and 2.5% were overweight. After six months, the proportion of children with a normal BMI rose by 7.5%, and the cases of underweight and overweight decreased by 2.5% and 5%, respectively. However, after one year, the BMI distribution returned to baseline levels, without any children with obesity at any time point. Similar findings were reported by Mager et al. [41]. Researchers observed that the majority of children were growing at age-appropriate rates at both diagnosis and the 1-year follow-up. However, two of them had low height-for-age and weight-for-age z-scores at diagnosis, which improved in one child after one year.

In contrast, Forchielli et al. [36] found that BMI increased from (mean ± standard deviation) 17.05 ± 4.3 kg/m² to 17.6 ± 4.7 kg/m^2^ in the long term, while waist-to-height ratio rose from 0.46 ± 0.06 to 0.47 ± 0.06. Critical evaluation of the energy findings led the researchers to further evaluate anthropometric parameters in the participants, comparing participants who experienced an unintentional caloric restriction in the long term with those receiving more calories. At the end of the year, a reduced caloric intake caused growth retardation for height by 2 cm and for weight by 800 g, but this change did not differ significantly regarding weight. Moreover, children who consumed fewer calories weighed 1.3 kg less and were 2.6 cm shorter than their peers, according to a comparable study conducted on children ages 10 or under. Only children who started the GFD with a weight in the 50th percentile for their age, however, showed no changes.

Likewise, in a study by Sila et al. [44], body weight, body height, and BMI z-scores of children with CD at diagnosis were compared to those recorded after an average of 34.1 months on a GFD. Significant increases were observed in all three parameters: the mean body weight z-score increased from –0.4 ± 1.3 to 0.2 ± 1.0, the mean body height z-score increased from –0.2 ± 1.0 to 0.3 ± 1.1, and the mean BMI z-score increased from –0.3 ± 1.2 to 0.0 ± 1.0.

### 3.6. Associations of a GFD with Body Composition and Bone Mineral Density

With regard to body composition, one research group [33] assessed fat and muscle mass, two research groups [22,39] examined body fat percentage, while another two [22,41] focused on body mineral density.

Allowaymi et al. [33] reported that 54.1% of girls and 65.5% of boys had a normal fat mass, while 51.4% of girls and 62.1% of boys had a normal muscle mass. Larretxi et al. [39] revealed that body fat percentage was adequate in most children and adolescents with CD. Specifically, there were no differences between genders from 3 to 13 years, but at age 14–18, body fat percentages were higher in girls than in boys, as expected (girls, 25.3 ± 5.8 vs. boys, 13.6 ± 9.2, *p* < 0.001). Ballestero Fernández et al. [22] showed no association between body fat percentage in children with CD and HCs. Remarkably, according to established cut-off points, more than 62% of children in both groups were categorized as having a normal body fat percentage.

Low bone mineral density, one of the primary risk factors for fractures in children [54], can be affected by the presence of untreated CD [55]. The underlying mechanisms mediating the malabsorption of Vitamin D and calcium are attributable to the T-cell-mediated inflammatory process resulting in villus atrophy of the small intestine mucosa.

At the time of diagnosis of CD, children and adolescents have suboptimal bone health and shorter stature, which suggests that young adults may experience a higher prevalence of reduced BMD [56]. The risk of osteoporosis later in life may be raised by inadequate bone development at the crucial time of peak bone mass formation, which is usually between the ages of 20 and 30. Interestingly, a long-term GFD can improve BMD in children and adolescents with CD [57]. In the present systematic review, there were no studies assessing short-term effects of GFD on BMD, whereas only two research groups evaluated BMD in the long-term.

Specifically, Mager et al. [41] investigated the relationships between vitamin K and D status and lifestyle factors on BMD in children and adolescents with CD at the time of diagnosis and after one year on a GFD. Whole-body and lumbar spine BMD were assessed using a dual-energy X-ray absorptiometry (DXA) scan. The WHO standards were applied to define BMD, with a BMD-z score ≤ −2.0 indicating osteoporosis. For the purposes of the analysis, BMD-z scores ≤ −1.0 were defined as increased risk for poor bone health. Researchers showed that whole-body and lumbar-spine BMD-z scores were low both at diagnosis (10–20%) and after a long-term adherence to the GFD (30–32%). Additionally, whole-body BMD z-scores were significantly lower in children > 10 years old compared to younger children (0.55 ± 0.7 vs. 0.72 ± 1.5, respectively, *p* < 0.001). Overall, the coexistence of suboptimal vitamin D and K and calcium levels, along with insufficient dietary intake of vitamin D and calcium, may contribute to an increased risk for poor bone health.

In contrast, Ballestero Fernández et al. [22] conducted a complete nutritional assessment in patients and healthy children and adolescents. Participants with CD were following a long-term GFD. BMD was measured using an ultrasound bone densitometer applied to the calcaneus. No differences in BMD were observed between children with CD and adolescents and HCs. Interestingly, participants with CD did not meet Vitamin D and calcium dietary recommendations for the Spanish population, while median 25-OH vitamin D plasma concentrations were within a mildly deficient range. Nevertheless, no relationship was found between BMD and vitamin D levels.

Overall, there were only a limited number of studies investigating body composition in children and adolescents with CD following a GFD. The results show a normal body fat percentage and conflicting results regarding BMD. Further observational studies are needed to investigate the relationship between GFD and body composition in the pediatric CD population.

### 3.7. Associations of a GFD with Food Habits

A variety of naturally gluten-free (GF) food groups, including meat, legumes, dairy products, fruits, vegetables, and oils, are included in the GFD [26]. However, current evidence suggests that gluten elimination often leads to suboptimal dietary choices by children and adolescents with CD, with a significant preference for high-fat and protein meals [35]. Moreover, the GFD combines the naturally occurring GF foods with GF products (GFPs) of cereal-based foods [24], whose nutritional profile has been subject to criticism within the scientific community [58]. Understanding how children and adolescents with CD integrate food groups and GFPs into their diet is essential for ensuring optimal nutritional status.

In this context, Allowaymi et al. [33] evaluated the nutrient intake of children with CD who followed a 6-month GFD program. Researchers showed that patients were following a carbohydrate-rich diet, primarily due to the GFD offered by the Saudi Ministry of Health, which consists of flour, bread, pasta, oats, crackers, and cornflakes. Additionally, typical main courses for lunch and dinner often consisted of high-carbohydrate GF products, including pasta or macaroni and rice with meat, further contributing to the carbohydrate-rich diet.

Zuccotti et al. [46] identified differences in eating habits between children with CD following a GFD for a short term and HCs. The results show that children with CD consumed more bread, rice, and low-nutrient foods including junk food, biscuits, and soft drinks compared to the HCs. Pasta consumption was comparable between the two groups, with no statistically significant differences.

Concerning the long-term adherence to a GFD, Ballestero Fernández et al. [22] did not observe any significant differences in the food group consumption between children and adolescents with CD and HCs. An exception was shown in patients consuming lower amounts of cereals and higher amounts of fruit (only in male patients) compared to their healthy peers. Additionally, Larretxi et al. [39] assessed the eating habits of children and adolescents with CD. The results show that while most children and adolescents with CD incorporated cereal products into their breakfast, only 33% of them consumed the recommended four servings per day. Around 21% ate pasta or rice almost daily, and two out of ten consumed fewer than two servings of cereals. Moreover, 86% of the participants did not reach the vegetable consumption recommendations. The majority of participants included one fruit or fruit juice per day; however, nearly half of them included a second serving of fruit daily. Regarding animal-origin food, 73% of participants consumed dairy products daily, with two out of ten children and adolescents with CD exceeding four servings daily. Meat consumption was excessive in 64%, while 50% and 42% reached the recommended amounts of fish and eggs, respectively. As mentioned above (see Section 3.2), an imbalance in lipid intake was observed, with the GFD showing lower levels of MUFA and PUFA but higher levels of SFA compared to the GCD. This is largely attributed to the tendency of children to replace plant-based foods with animal products. 80% of participants reached the recommended amount for pulses (2–4 portions/week); however, only 28% consumed nuts on a daily basis. Finally, vegetable oil, sugar, chocolate, and pastry consumption was in line with recommendations.

Lionetti et al. [40] evaluated food group consumption of children and adolescents with CD compared to HCs and assessed whether it aligned with the recommendations of the “Italian Food Pyramid” (IFP) established by the Italian Society of Pediatrics. The results show that children with CD consumed more processed meat and salty snacks than HCs (2.5 portions in the CD group against 2 in the control group; *p* = 0.009, and 1 portion against 0; *p* = 0.001, respectively). Both groups consumed higher amounts of sugary beverages, meat, and processed meat than IFP guidelines, while they did not fulfill the recommendations regarding legumes, vegetables, eggs, and fish. Notably, fruit consumption was above the IFP’s minimum recommended intake, and cereal, dairy products, and potato intake aligned with IFP requirements. Interestingly, the CD group’s consumption of pseudo-cereals was significantly lower than that of the cereal group, with GFPs designed especially for CD accounting for the majority of the consumption.

As far as GFPs are concerned, the included studies indicate that GFPs constitute a significant portion of the total daily energy intake, contributing in a range between 36.3% [46] and 46% [40] of energy. Additionally, Zuccotti et al. [46] observed that 77.0% of carbohydrate-derived energy came from GFPs. Notably, in a study by Ballestero Fernández et al. [22], although participants reported consuming processed GFPs from two to three times per day, they declared having sufficient nutritional education regarding CD and product labeling and considered themselves capable of choosing proper food products.

## 4. Discussion

### 4.1. Summary of the Primary and Secondary Outcomes

In the present systematic review, we meticulously examined 15 observational studies to determine the efficacy of a short-term (≥6 months to <12 months) [33,42,46] and long-term (≥12 months) [22,34,35,36,37,38,39,40,41,43,44,45] adherence to a GFD on nutritional status in children and adolescents diagnosed with CD. To our knowledge, this is the first systematic review that comprehensively analyzes both short- and long-term nutrient intake in children and adolescents with CD following a GFD, also highlighting changes in anthropometric characteristics, body composition, and food habits of the patients over time. However, the majority of the studies included are of low quality, since 3/15 showed “some concerns”, 3/15 showed “high” risk, and 7/15 showed “very high” risk of bias according to the tools used to assess the risk of bias (ROBINS-E tool) [17,19,45,47,55,56].

Despite the methodological heterogeneity among the included studies, certain consistent patterns emerge regarding nutrient intake in the pediatric population with CD. Results from the cross-sectional/case–control studies in the short term show that children and adolescents with CD consumed excessive amounts of protein and carbohydrates compared to controls. The most frequently observed micronutrients with inadequate diet intake included vitamin D, calcium, folic acid, and magnesium. Notably, only one study assessed B-complex vitamins and selenium, reporting lower intake in vitamins B1, B2, B3, and B6 compared to DRI [33] and inadequate selenium intake compared to HCs [46]. Zinc dietary intake was consistently below recommended levels for all children and adolescents with CD, regardless of whether they adhered to a gluten-free diet. Regarding vitamin C, one study reported insufficient intake [38], while another noted deficiencies specifically among girls [33].

After long-term adherence to a GFD, significant changes in the diet of children and adolescents with CD persist. Fat intake is higher, while protein intake remained excessively high compared to controls, with both macronutrients frequently exceeding dietary guidelines. While there are some improvements in some micronutrients, deficiencies in key nutrients, including vitamin D, calcium, iron, folic acid, magnesium, and zinc, remain widespread. However, many studies suggest that these nutrient deficiencies are prevalent in children and adolescents, regardless of whether they follow a GFD. However, vitamin C intake tends to normalize over time.

Based on prospective cohort studies, many patients already have significant nutritional deficiencies at the time of diagnosis, including an inadequate supply of iron, calcium, vitamins B1, B2, B6, C, D, folate, selenium, and iodine. While intakes of vitamin C and iodine improved both in the short and long term after adherence to a GFD, intakes of most other nutrients either remained inadequate or continued to decline, indicating that, despite dietary adjustments, it remains difficult to meet nutrient requirements.

The biochemical analysis revealed a persistent deficiency of blood vitamin D levels, which frequently fell below the normal range. However, this deficiency is not unique to children with CD on a GFD, as similar trends are also seen in HC children. Despite the low dietary iron intake, blood iron levels generally remained stable.

In terms of secondary outcomes, GFD appears to be negatively associated with height in children and adolescents with CD in both the short and long term. Regarding body weight and BMI status in the short term, research is limited. The only existing study showed a similar growth pattern in patients with CD compared to the general pediatric population. However, findings from long-term case–control studies are mixed. Two research groups [44,45] found a higher prevalence of both underweight and overweight in children and adolescents with CD compared to HCs [44,45], while, in contrast, another study reported higher percentages of normal BMI in patients with CD compared to HCs. Prospective studies tracking growth from diagnosis to 12 months on a GFD show more consistent results. These studies indicate that BMI (or BMI z-score) tends to improve over time, moving toward healthier levels.

Regarding body composition, studies focused only on children who had followed a GFD for a long-term period. Body fat percentage was aligning with the normal values in children and adolescents with CD, while researchers supported the idea that GFD may affect negatively BMD, as deficiencies in key nutrients like calcium and vitamin D can impact skeletal development.

Finally, GFPs contribute significantly to daily energy intake, primarily as a source of carbohydrate-derived calories.

### 4.2. Literature Documentation

All patients with CD are advised to strictly adhere to a GFD, which is regarded as a crucial component of patient care [59], leading to an improved intestinal absorption that occurs as a result of mucosal healing induced by a GFD [16]. In line with the present review, numerous studies in both adults and children diagnosed with CD have indicated nutritional deficiencies as a consequence of GFD [17,19,49,52,60]. Importantly, we have found that many of these dietary imbalances persist in the long term. Although outside the scope of this systematic review, correction of nutritional deficiencies through supplementation is recommended for the initial few months and may be performed over a long period of time given the restricted nature of GFDs [61]. Along similar lines, ESPGHAN highlights the potential effects of supplementation alongside a GFD in children and adolescents with CD and anemia due to iron, folate, and vitamin B12 deficiency.

The transition from naturally gluten-containing foods to gluten-free grain alternatives often leads to a deterioration in nutritional quality due to their lower contents of fiber, protein, and important vitamins and minerals [62,63]. Frequent consumption of processed GFPs, as confirmed in the present systematic review, may further contribute to these dietary imbalances. Indeed, when comparing GFPs with their gluten-containing counterparts, a poorer nutrient profile was found in terms of both macro- and micronutrients [58,62,63,64].

From this perspective, these dietary changes have been identified as potential risk factors for overweight and obesity in children and adolescents with CD [29,65]. The rising prevalence of overweight and obesity in pediatric populations may also contribute to weight gain observed in patients with CD [66]. CD is associated with abnormalities in the metabolism of fat and carbohydrates [67]. It has been shown that the oxidation of carbohydrates is favored in untreated celiac disease, which is most likely due to the malabsorption of lipids and increased carbohydrate intake [67,68]. Furthermore, in patients not receiving treatment, fecal lipid loss is positively correlated with high rates of carbohydrate oxidation. GFD significantly increases body fat stores by increasing lipid usage when the intestinal mucosa is restored [67,69]. On the other hand, inadequate nutrient intake can lead to growth failure due to short stature and can lead to poor weight gain in children and adolescents with CD [70,71]. In line with the existing literature, our results on growth in patients with CD remain inconsistent.

Current evidence suggests that a normal BMD is achieved with a long-term adherence to a GFD [57,58]; however, the present systematic review shows contradictory results, since, in one study, BMD was greatly reduced after 12 months on a GFD [41], which may cause difficulties in achieving peak bone mass [58]. Vitamin D is essential for calcium absorption and bone mineralization, which is positively associated with bone mineral density [72,73]. Epidemiological studies generally support the finding that a GFD is associated with adequate blood levels of Vitamin D and calcium [74,75,76,77]. As already mentioned, the results of the current systematic review suggest a general blood vitamin D deficiency, which coincides with a worldwide vitamin D deficiency affecting from 10 to 50% of the pediatric population. Finally, even though iron deficiency anemia is among the most prevalent laboratory abnormalities observed in patients with CD [18], most studies show that blood iron levels were within normal ranges independent of low dietary iron intake.

In addition, the socio-economic conditions, regional variability, age-related factors, and the existing comorbidities of the studied populations may contribute to the inconsistency observed across studies. Urban living is associated with better GFD adherence due to access to GFPs, healthcare services, and dietary interventions [78]. Higher income levels are associated with improved dietary quality and better access to gluten-free foods, while patients from lower-income backgrounds face challenges in adhering to a GFD due to limited sources and availability of suitable gluten-free options [79,80]. Heterogeneity across the included studies is strongly associated with regional variations. The prevalence of celiac disease varies across Europe, from 0.3% in Germany to 2.4% in Finland, while higher rates are observed in developing regions, particularly in North Africa and the Middle East [81]. Furthermore, adherence to a GFD tends to decline during adolescence [82] due to social pressures, the desire for normalcy, and increased independence in food choices [83], making younger children more likely to eliminate gluten from their diet. Additionally, celiac disease is frequently diagnosed alongside type 1 diabetes, presenting further challenges for nutrient intake since gluten-free foods have high glycemic index, while low glycemic index foods are recommended for managing type 1 diabetes mellitus [84].

Up until today, there are no published recommendations for the follow-up of children diagnosed with CD [85,86], with the current literature offering limited high-quality evidence to guide the frequency and structure of follow-up appointments [85]. According to the ESPGHAN position paper [85], the first follow-up visit is recommended 3–6 months after diagnosis. In addition, follow-up appointments should initially be arranged every 6–12 months and then every 12–24 months for long-term treatment. Individual circumstances such as ongoing symptoms, family concerns, or the presence of malnutrition and laboratory abnormalities at the time of diagnosis should be considered when scheduling follow-up appointments. In a similar way, NASPGHAN highlights the importance of a multidisciplinary approach involving pediatric gastroenterologists and CD-experienced dietitians [86]. Evaluation of dietary adherence, treatment of vitamin deficiencies, and management of persistent symptoms all depend on routine follow-up, especially in the first year or two.

### 4.3. Strengths and Limitations

This present systematic review has some important strengths and possible limitations. One key aspect of this systematic review is that the current methodology was based on the high-quality standards of the Preferred Reporting Items for Systematic Reviews and Meta-Analyses (PRISMA) [31]. Of the 15 selected articles, almost half were published after 2020 and therefore provided up-to-date results. In addition, this review addresses a wide range of nutritional and anthropometric outcomes related to CD and adherence to a GFD. It provides a thorough analysis of short- and long-term effects, considering deficiencies and imbalances across a spectrum of nutrients to provide a holistic view of the nutritional challenges associated with CD and adherence to the GFD.

Several limitations were identified in the preparation of this review. In particular, there was a lack of high-quality observational studies, as the dietary assessments were based on self-reports, while adherence to a GFD was not assessed. A high dependence on dietary self-assessment was noted, which may have influenced the results in different ways. Common sources of error in self-reported dietary assessments included omissions and inaccuracies in estimating portion sizes of food and beverage items [87]. In addition, FFQs, which are widely used in nutritional epidemiology, often overestimate macro- and micronutrient intake compared to more direct methods such as food diaries and 24 h recalls [88,89]. In one third of the studies, adherence to a GFD was not verified by serological testing. Long-term consumption of gluten, even in minimal amounts, can contribute to chronic inflammation and malabsorption, with gluten-related manifestations affecting various organs, particularly growth [18].

As mentioned above, methodological discrepancies were found both between the categories of short- and long-term dietary patterns and within these categories, particularly in relation to the comparator used (i.e., HCs, DRI, RDA, EFSA recommendations, time of diagnosis). It was therefore not possible to conduct a meta-analysis due to the substantial differences in research designs.

## 5. Conclusions

In the present study, 15 observational studies were systematically reviewed to identify critical periods of dietary imbalance and support the development of tailored nutritional strategies to mitigate the long-term health risks associated with a GFD in pediatric populations. Despite the lack of high-quality observational studies and considerable methodological heterogeneity, our findings emphasize the need for early and continuous nutritional assessments to support optimal growth and health outcomes. At the time of CD diagnosis, a comprehensive nutritional assessment with laboratory analysis and anthropometric measurements should be performed to identify any nutritional deficiencies and address them with targeted interventions until normalization occurs. Regardless of the presence of nutritional deficiencies, trained dietitians should provide nutrition education to patients and their caregivers to promote adherence to a high-quality nutrient-rich GFD. Follow-up should begin 6 months after diagnosis to reassess nutrient intake and growth, and be performed annually thereafter, as continued counseling ensures a balanced GFD. High-quality observational studies with standardized methodology should be a priority in future research to provide robust information for the development of nutritional guidelines and long-term strategies for children and adolescents with CD.

## Figures and Tables

**Table 1:** Risk of bias assessment.

Study (Author, Year)	Risk of Bias							
	D1	D2	D3	D4	D5	D6	D7	Overall
Allowaymi et al., 2022 [33]	−	+++	−	−	+	++	+	+++
Balamtekin et al., 2015 [34]	+	++	−	−	−	++	−	++
Ballestero Fernández et al., 2019 [22]	+	−	−	−	−	−	−	+
Ferrara et al., 2009 [35]	+++	+++	++	+	−	++	++	+++
Forchielli et al., 2015 [36]	−	−	−	−	−	−	−	−
Forchielli et al., 2019 [37]	−	−	−	−	−	−	−	−
Kozioł-Kozakowska et al., 2015 [38]	++	+++	−	+	+++	+	−	+++
Larretxi et al., 2018 [39]	−	+++	−	−	++	−	−	+++
Lionnetti et al., 2020 [40]	+	+	−	−	+	−	−	+
Mager et al., 2011 [41]	+	−	+	+	++	−	−	++
Nestares et al., 2020 [42]	−	+++	−	+	+	−	−	+++
Rujner et al., 2003 [43]	+	−	−	+	++	−	−	++
Sila et al., 2022 [44]	++	+++	−	++	+++	+	−	+++
Szaflarska-Popławska et al., 2022 [45]	+	+	−	−	+++	+	−	+++
Zuccotti et al., 2012 [46]	+	+	+	−	−	−	−	+

Di = bias domain, i = 1–7 as follows: D1: due to confounding; D2: arising from measurement of the exposure; D3: in selection of participants in the study; D4: due to post-exposure interventions; D5: due to missing data; D6: arising from measurement of the outcome; D7: in selection of the reported result. Symbol used for risk of bias: −low risk of bias; + some concerns; ++ high risk; +++ very high risk.

**Table 2:** Summary of the findings on the association between GFD and energy or/and macronutrient intake.

Study	Type of study	Population			Duration of GFD	Results				
		N	Age ^a^	Sex ^b^		Energy	Protein	Carbohydrate	Fat	Fiber
Allowaymi et al., 2022 [33]	Cross-sectional	66 (CD)	10.4 ± 4.1 y (6–16 y)	F: 37 M: 29	Short term ≥6 months	↓ (*p* ≤ 0.001) (compared to DRI) G: 1296 kcal B: 1400 kcal DRI: 1800 kcal	↑ (*p* ≤ 0.001) (compared to DRI) G: 42.94 g B: 47.37 g DRI: 34 g	↑ (*p* ≤ 0.001) (compared to DRI) G: 172 g B: 192.8 g DRI: 130 g	NS	↓ (*p* ≤ 0.001) (compared to DRI) G: 12.85 g B: 12.51 g DRI: 25 g
Nestares et al., 2020 [42]	Cross-sectional case–control study	68 (CD)	8.5 ± 4.1 y (7–18 y)	F: 52 M: 16	Short term >6 months	NS	NS	NS	NS	NS
		43 (HC)	10.3 ± 4.5 y	F: 20 M: 23						
Zuccotti et al., 2012 [46]	Cross-sectional case–control study	18 (CD)	7.6 ± 2.8 y (4–10 y)	G: 13 B: 5	Short term ≥6 months	↑ (*p* < 0.001) (compared to HCs) CD: 2141.92 ± 680.44 kcal HC: 1376.91 ± 1009.6 kcal	↑ (*p* = 0.012) (compared to HCs) CD: 68.3 ± 22.7 g HC: 55.7 ± 30.0 g	↑ (*p* < 0.001) (compared to HCs) CD: 286.6 ± 88.6 g HC: 149.1 ± 62.1 g	NS	NS
		18 (HC)	7.0 ± 2.3 y	F: 11 M: 7						
Ballestero- Fernández et al., 2019 [22]	Cross-sectional age and gender-matched study	70 (CD)	(4–18 y)	F: 35 M: 35	Long term ≥1 year	-	↓ (*p* < 0.05) (compared to HCs) CD: 15.5% TEI HCs: 16.5% TEI	NS	NS	NS
		67 (HC)		F: 26 M: 41						
Balamtekin et al., 2015 [34]	Cross-sectional case–control study	28 (CD)	10.3 ± 4.6 y (3–18 y)	F: 22 M: 6	Long term 4.0 ± 3.3 y	↓ (*p* = 0.002) (compared to HCs) CD: 1582.7 ± 419.4 Kcal HC: 1921.8 ± 321.2 kcal	↓ (*p* < 0.002) (compared to HCs) CD: 45.5 ± 12.9 g HC: 6.3 ± 12.5 g	↓ (*p* = 0.002) (compared to HCs) CD: 190.0 ± 68.2 g HC: 244.8 ± 53.4 g	NS	↓ (*p* = 0.001) (compared to HCs) CD: 13.8 ± 7.0 g HC: 20.1 ± 5.7 g
		25 (HC)	9.5 ± 3.4 y	F: 18 M: 7						
Ferrara et al., 2009 [35]	Retrospective	50 (CD)	10.68 y (6–16 y)	F: 32 M: 18	Long term ≥1 year	NS	-	-	↑ (*p* < 0.008) (compared to HCs) CD: 72.5 ± 37.2 g HC: 52.9 ± 35.4 g	-
		50 (HC)	10.74 y (6–17 y)	F: 33 M: 17						
Forchielli et al., 2015 [37]	Longitudinal study	205 (CD)	10.7 ± 4.2 y (1–18 y)	F: 132 M: 73	Long term 6.2 ± 4.1 y	↓ (*p* ≤ 0.0001) (compared to LARN) CD: 1761.6 ± 453.6 kcal LARN: 2054.5 ± 568.4 kcal	↑ (*p* ≤ 0.0001) (compared to LARN) CD: 66.2 ± 23.8 g LARN: 29 ± 12 g	NS	NS	NS
Larretxi et al., 2018 [39]	Cross-sectional	83 (CD)	9.2 ± 3.8 y (3–18 y)	F: 53 M: 30	Long term 6.43 ± 4.18 y	NS	↓ (*p* ≤ 0.001) (compared to GCD) GF:79.8 ± 17.0 g GC:83.5 ± 17.2 g	↓ (*p* ≤ 0.001) (compared to GCD) GF:219.0 ± 47.1 g GC:223.2 ± 42.2 g	↑ (*p* ≤ 0.001) (compared to GCD) GF:86.2 ± 19.6 g GC: 83.2 ± 19.5 g	-
Lionnetti et al., 2020 [40]	Case–Control Prospective Study	120 (CD)	10.5 (4.4–15.5 y)	F: 72 M: 48	Long term ≥2 years	NS	NS	↓ (*p* = 0.001) (compared to HCs) CD:209.7 ± 25.6 g HC:260.5 ± 18.3 g	↑ (*p* = 0.015) (compared to HCs) CD: 78.1 ± 7.3 g HC: 64.4 ± 5.2 g	↓ (*p* = 0.015) (compared to HCs) CD: 12.6 ± 1.5 g HC: 15 ± 1.9 g
		100 (HC)	10.1 (4.7–14.5 y)	F: 56 M: 44						
Sila et al., 2022 [44]	Cross-sectional case–control study	76 (CD)	9.0 ± 4.3 y	F: 43 M: 33	Long term 34.1 ± 25.4 y	↑ (*p* < 0.001) (compared to HCs) CD: 1740.3 ± 482.2 kcal HC: 1454.8 ± 423.9 kcal	↑ (*p* = 0.021) (compared to HCs) CD:67.0 ± 19.4 g HC:62.1 ± 17.1 g	-	↑ (*p* < 0.001) (compared to HCs) CD: 70.0 ± 26.7 g HC: 52.4 ± 19.7 g	NS
		590 (HC)	9.9 ± 0.1 y (6–14 y)	F: 317 M: 273						
Szaflarska-Popławska et al., 2022 [45]	Single-center prospective cohort study	48 (CD)	11.8 ± 3.6 y	F: 33 M: 15	Long term 5.02 ± 3.87 y	NS In line with the reference values	NS 190.3% DRI	NS 189.4% DRI	NS In line with the reference values	NS In line with the reference values
		50 (HC)	10.2 ± 3.8 y	F: 26 M: 24						
Rujner et al., 2004 [43]	Cross-sectional case–control study	41 (CD)	5.9–18.3 y	F: 29 M: 18	Long term 2.7–17.3 y	↓ (compared to HCs) 139.2% RDI (CD) 150.5% RDI (HCs)	↓ (compared to HCs) 113% RDI (CD) 141.2% RDI (HCs)	-	↑ (compared to HCcs) 163% RDI (CD) 153.8% RDI (HCs)	-
		8 (HC)	6.4–17.3 y	F: 4 M: 4						
Forchielli et al., 2019 [37]	Prospective	79 (CD)	7.9 ± 3.8 y (1–18 y)	F: 52 M: 27	Diagnosis (T0) Short term 6 months (T1) Long term 12 months (T2)	↓ at diagnosis ↓↓ after 12 months (*p* ≤ 0.001) (compared to recommendations) T0: 1786 ± 401.8 kcal T2: 1698.33 ± 377.46 kcal Recommendations: 1925.9 ± 504.1 kcal	↑ diagnosis ↑ 12 months (*p* < 0.001) (compared to recommendations) T0: 65 ± 21.9 g T2: 62 ± 19.3 g Recommendations: 26.2 ± 10.5 g	NS	NS	↑ 12 months (*p* = 0.046) (compared to time of diagnosis) T0: 12.2 ± 4 g T2: 13.1 ± 5.2 g
Mager et al., 2011 [41]	Prospective	43 (CD)	9.4 ± 4.2 y (3–17 y)	F: 30 M: 13	Diagnosis (T0) Long term 12 months (T2)	↓ (*p* < 0.05) (after 12 months) T0: 1813 ± 680 kcal T2: 1611 ± 318 kcal	↓ (*p* < 0.05) (after 12 months) T0: 61.5 ± 27.2 g T2: 56.7 ± 10.8 g	-	-	-
Kozioł-Kozakowska et al., 2015 [38]	Prospective	40 (CD)	8.4 ± 3.5 y (7–17.5 y)	F: 28 M: 12	Diagnosis (T0) Short term 6 months (T1) Long term 12 months (T2)	↑ after 6 mo ↑↑ after 12 mo (*p* = 0.02) (compared to time of diagnosis) T0: 1440.59 ± 367.44 kcal T1: 1551.30 ± 384.14 kcal T2: 1629.11 ± 368.09 kcal No significant difference compared to EER	NS	NS	NS	NS

^a^ Presented as mean ± standard deviation or median (1st, 3rd quartile) and/or range ^b^ Presented as absolute (relative) frequency. Abbreviations: CD: celiac disease, DRI: dietary reference intake, EER: estimated energy requirements F: Female, GCD: gluten-containing diet HCs: healthy controls, M: Male, mo: months, NS: Not significant, *p*: *p*-value, RDI: recommended daily intake, TEI: Tota lEnergy Intake (%), y: year(s).
